# Eradication of HCV Infection with the Direct-Acting Antiviral Therapy in Renal Allograft Recipients

**DOI:** 10.1155/2019/4674560

**Published:** 2019-04-07

**Authors:** Calogero Armando, Sagnelli Evangelista, Creta Massimiliano, Angeletti Silvia, Peluso Gaia, Incollingo Paola, Candida Maria, Minieri Gianluca, Carlomagno Nicola, Dodaro Concetta Anna, Ciccozzi Massimo, Sagnelli Caterina

**Affiliations:** ^1^Department of Advanced Biomedical Sciences, University of Naples Federico II, Naples, Italy; ^2^Department of Mental Health and Public Medicine, University of Campania Luigi Vanvitelli, Naples, Italy; ^3^Department of Neurosciences, Human Reproduction and Odontostomatology, University of Naples Federico II, Naples, Italy; ^4^Unit of Clinical Laboratory Science, University Campus Bio-Medico of Rome, Rome, Italy; ^5^Unit of Medical Statistic and Molecular Epidemiology, University Campus Bio-Medico, Rome, Italy

## Abstract

Hepatitis C virus (HCV) infection unfavorably affects the survival of both renal patients undergoing hemodialysis and renal transplant recipients. In this subset of patients, the effectiveness and safety of different combinations of interferon-free direct-acting antiviral agents (DAAs) have been analyzed in several small studies. Despite fragmentary, the available data demonstrate that DAA treatment is safe and effective in eradicating HCV infection, with a sustained virologic response (SVR) rates nearly 95% and without an increased risk of allograft rejection. This review article analyzes the results of most published studies on this topic to favor more in-depth knowledge of the readers on the subject. We suggest, however, perseverating in this update as the optimal DAA regimen may not be proposed yet, because of the expected arrival of newer DAAs and of the lack of data from large multicenter randomized controlled trials.

## 1. Introduction

Hepatitis C virus (HCV) infection affects nearly 150 million people worldwide and more than 350,000 people die each year of HCV-related diseases [1–7]. In the adult population, HCV infection is associated with an increased risk of developing chronic kidney disease [8] and the prevalence of HCV infection is greater among patients with chronic kidney disease (1.8% -8%) than in the general population (0.5-4%), especially in those undergoing hemodialysis and in renal allograft recipients [9–22]. In these patients, HCV infection is a risk factor for other comorbidities [9, 23, 24]. In fact, in renal allograft recipients, HCV has been found to be associated with posttransplant proteinuria, with an increased risk of new-onset diabetes possibly leading to cardiovascular diseases and malignancies, and with allograft loss, infections, and death [9, 12, 25–40]. Therefore, it is highly desirable to treat HCV infection before or after renal transplant to eliminate at the same time both HCV infection and the risk of complications [40–50].

Interferon-based regimens had been the backbone of HCV treatment for kidney transplant recipients until 2014, a therapeutic approach limited by the relatively low efficacy in achieving viral eradication, poor tolerability [51, 52], and the obligation to use it only before transplant because of the high risk of inducing immune stimulation and allograft rejection [53–55].

The first-generation direct-acting antivirals (DAAs) protease inhibitors telaprevir and boceprevir present a similar limitation because their efficacy depends on the coadministration of interferon [56–63]. Instead, the currently available new generation DAAs, associated with a greater sustained viral response (SVR) and with a good safety profiles, have strongly improved the treatment of chronic HCV infection even in renal patients [64–68]. HCV eradication is testified by the achievement of a sustained virologic response 12 (SVR12), highlighted by the undetectability of serum HCV RNA within the 12^th^ week of treatment and throughout a subsequent follow-up of 12 weeks.

A clear example of the efficacy of DAAs in eradicating HCV infection in patients with chronic kidney diseases (CKD) is offered by the C-SURFFER study, a multicenter, double-blind, randomized study where 224 patients with HCV GT1 infection and CKD stages 4 or 5 were randomly assigned to an “immediate treatment group” receiving grazoprevir and elbasvir (n=111) for 12 weeks or to a “deferred treatment” group treated with the same schedule (n=113) 16 weeks later. Most of patients were haemodialysis dependent; half of them had HCV genotype 1a infection, 80% were HCV treatment naïve, and 6% had liver cirrhosis. SVR rates in the “immediate treatment” and “deferred treatment” groups were 99% and 98, respectively [69].

There are four classes of direct-acting antiviral drugs that combine the attack on more than one HCV life cycle goal: (1) NS3/4A protease inhibitors (galexos, grazoprevir, sunvepra, glecaprevir, paritaprevir, and voxilaprevir); (2) the nucleoside and nucleotide NS5B polymerase inhibitors (sofosbuvir); (3) NS5A inhibitors (ombitasvir, pibrentasvir, daclatasvir, elbasvir, ledipasvir, ombitasvir, and velpatasvir); and (4) nonnucleoside NS5B polymerase inhibitors (dasabuvir).

General symptoms (adverse events, fatigue, nausea, dizziness, or headache) associated with DAA treatment in kidney transplant recipients have been reported in nearly 40% of treated patients, whereas severe adverse events (i.e., anemia, portal vein thrombosis, or pneumonia) are infrequent events (around 1% of treated patients) [70].

Glomerular disease after DAAs has been also described. Lubetzky et al. [71] reported no significant change in glomerular filtration rate (GFR) before or after therapy, but 3 patients showed a decrease of GFR to less than 20 and 19.3% of 31 patients had worsening proteinuria during or shortly after therapy. Besides, patients with a proteinuria over 500 mg/g before treatment showed an increased value during treatment more frequently than those with initial values lower than 500 mg/g (P < 0.001) [71].

In this review article we analyze the data from several real-life studies on the feasibility, effectiveness, and tolerability of DAA therapies for kidney transplant recipients. The experience of most single studies is limited, prevalently by the low number of patients examined, but a comprehensive analysis of the available data can give useful indications to the readers [68–75].

Episodes of reactivation of an indolent (open or occult) Hepatitis B virus (HBV) infection in patients with HBV/HCV-related chronic hepatitis during or after a successful DAA therapy have been documented [76]. HBV reactivation of open or occult HBV infection has been observed even in kidney-transplant recipients with HBV/HCV-related chronic hepatitis after a SVR had been achieved with DAA treatment. [77].

## 2. DAA Agents for Treatment of HCV Infection in Patients with Chronic Kidney Disease

In HCV-related liver diseases, the combined use of two or more second-generation DAAs allows the eradication of HCV infection in nearly 95% of treated patients [78–83], a percentage obtained even in liver transplant recipients [64, 66].

The knowledge on HCV eradication with DAA therapy in HCV-positive renal recipients or in those on a waiting list for renal transplantation is fragmentary, but the published data are highly promising. For HCV-positive patients in the wait list for kidney transplantation the first decision is whether to treat them before or after transplantation. For HCV patients with mild or moderate hepatic fibrosis who have a living kidney donor, treatment with DAA has been suggested prior to kidney transplantation [55, 67]. For HCV-positive patients who do not have a living donor and are at risk of progression to a more severe liver disease it has been suggested to perform kidney transplantation and to treat them with DAAs soon after, a decision that allows receiving a kidney from an HCV-positive donor [68] and reducing wait time for transplantation dramatically. Indeed, it has been recently reported that patients who accepted to be transplanted with an HCV-positive kidney, compared with those who have reclaimed an HCV-negative donor kidney transplant, have lower average waiting (469 vs 856 days) [67]. For an exhaustive analysis, it should be also considered that, due to the shortage of transplantable kidneys, in 2014, of 98, 956 patients on waiting list for kidney transplantation in the USA, more than 8,000 (8%) had died or were removed from the list due to worsening in clinical conditions [69, 75, 78].

All this considered, it is commonly accepted that HCV-infected transplant candidates may receive a kidney from an HCV-positive donor. This eventuality had occurred in about 2500 donations in the USA between 2005 and 2014 [73]; otherwise these kidneys would have been discarded. The transplantation of HCV-infected kidneys in HCV-infected patients, in addition to reducing waiting times [82], expands the pool of usable kidneys. The largest series [83] of renal transplants performed under these conditions demonstrated a 5-year and 10-year equivalent patient survival (p= 0.25) compared to those who received a kidney from an HCV-negative donor. Considering the persistent shortage of kidneys that can be transplanted, it has been also proposed to transplant HCV- positive kidneys into HCV-negative recipients and to initiate treatment with DAA immediately after transplantation and before a liver disease might develop.

At present, no valid recommendation comes from prospective trials, but several studies, prevalently small and from single clinical centers, provide useful information on the effectiveness and tolerability of different DAAs combination regimens in HCV-positive transplant recipients.

In a retrospective, multicentric study Fernández et al [84] observed a SVR12 in 98% of 103 kidney transplant recipients treated with DAAs (sofosbuvir plus ledipasvir in 59 and sofosbuvir plus daclatasvir in 18). Ribavirin was used in 1% of cases. Of these 103 patients, therapy was administered for 12 weeks in 72, for 24 weeks in 29, and for 8 weeks in two. There were three episodes of acute humoral graft rejection. No patient discontinued therapy due to adverse events, but 57 patients required immunosuppression dose adjustment. SVR was achieved by 98% of patients receiving DAAs alone and 97.6% of those receiving DAAs plus RBV, by 97% of those receiving 12 weeks of therapy, and 100% of those treated for 24 weeks. The presence of cirrhosis did not influence the response to treatment [84].

Lin et al. [85] treated 6 HCV-positive kidney recipients with a 12-week sofosbuvir/daclatasvir treatment (4 of 6 with HCV genotype 1b). HCV RNA was undetectable at week 4 and a SVR12 was obtained in all cases. Half dose of sofosbuvir was used in two patients with creatinine elevation. Treatment was well tolerated, and no adverse reaction had occurred.

Eisenberger et al. [83] assessed the efficacy and safety of 8- or 12-week combination of sofosbuvir plus ledipasvir in 15 kidney recipients with HCV infection (genotypes 1a, 1b, or 4). All patients achieved a SVR12, had a stable renal transplant function, and did not develop serious adverse events. A dose adjustment for tacrolimus was necessary in some cases. The authors concluded that the treatment was safe and effective.

Kamar et al. [66] treated 25 kidney transplant recipients with HCV infection with different sofosbuvir-based regimens. A rapid SVR, defined by undetectable viremia after 4 weeks of DAA therapy, was obtained in 22 (88%) cases and SVR12 in all. Treatments were well tolerated, and no adverse event had occurred, apart from a decrease in calcineurin inhibitor levels after HCV clearance. Lubetzky et al. [71] performed a retrospective cohort analysis of 31 HCV-positive patients who underwent kidney transplantation and received DAA therapy. The combination of sofosbuvir/ledipasvir was the most commonly used and 30 out of the 31 (97%) achieved SVR12; all patients showed a satisfactory allograft function. Six (19.3%) out of the 31 showed an increased proteinuria during or shortly after DAA therapy. The authors concluded that DAA therapy is safe and effective in HCV-positive kidney recipients and that patients with proteinuria should be monitored closely [71].

Bhamidimarri et al. [86] described 25 HCV patients in the end-stage kidney disease, transplanted with a kidney from an anti-HCV-positive deceased donor and treated with DAAs in the early post-transplant period (median 125 days). Of these 25, 24 (96%) achieved a SVR12. Tacrolimus dose adjustments were required in 13 patients.

Sawinski et al. [87] used 4 different DAA regimens to treat 43 renal transplant recipients; all of them obtained SVR 12 independently from the origin of the allograft (from an HCV-positive or -negative donor) and none of them showed a serious adverse reaction. A calcineurin inhibitor dose adjustment during treatment was applied in nearly half treated patients. Noteworthy, the waiting time to transplantation was longer in patients transplanted with a kidney from an HCV-negative donor than in those transplanted with an HCV-positive organ (969 versus 485 days) [84].

Colombo et al. [88] carried out a randomized, phase 2, open-label multicenter study including 114 kidney transplant recipients with HCV infection (HCV genotypes 1 or 4) infection and with a filtration rate (eGFR) of 40 mL/min or greater. These 144 were randomly assigned 1:1 to receive ledipasvir 90 mg and sofosbuvir 400 mg either for 12 or for 24 weeks; 91% had HCV genotype 1 infection and 15% compensated cirrhosis. A SVR12 was achieved by all treated patients, with no regard to the duration of treatment. Treatment was well tolerated in most cases, but serious adverse events occurred in 13 (11%) patients. The authors concluded that treatment with ledipasvir/sofosbuvir is effective ad shows an acceptable safety profile in HCV-positive kidney transplant recipients.

Londono et al. [89] described 103 HCV-positive kidney transplant recipients, 15% with cirrhosis, treated with DAAs, prevalently sofosbuvir/ledipasvir (n=59, 57%) or sofosbuvir/daclatasvir (n=18, 17%). Ribavirin was associated with DAAs in nearly half patients. The SVR12 rate was 98%. A grade 2 or 3 anaemia developed in 14 (33%) patients treated with ribavirin and in 9 (15%) untreated (p=0.03). No patient discontinued therapy because of an adverse event, but an adjustment of the dose of immunosuppressive drugs was required in nearly half of them. A mild allograft dysfunction occurred only in cirrhotic patients. An acute humoral graft rejection occurred in 3 patients. The authors concluded that DAA therapy was highly efficacious and safe in kidney transplant recipients.

Gallegos-Orozco et al. [90] described 8 HCV-positive kidney recipients (7 with an HCV-positive kidney and one with an HCV-negative kidney), treated three to six months after transplantation with DDA therapy according to HCV genotype and their prior treatment experience: all of them had functioning kidney grafts and achieved SVR12. The authors concluded that HCV-positive patients can successfully receive an HCV-positive kidney from an HCV-positive donor, with a substantial reduction of time on the wait list.

Durand et al. [91] conducted a nonrandomized open label study to evaluate the tolerability and efficacy of DAA treatment given before and after kidney transplantation to 10 non-HCV infected kidney recipients receiving an HCV-infected organ. All recipients transplanted with an HCV- positive genotype 1 infected kidney were treated with grazoprevir, 100 mg, and elbasvir, 50 mg, immediately before transplantation and for 12 weeks after transplantation, while for the recipients of a kidney from a donor infected with HCV genotypes 2 or 3 sofosbuvir, 400 mg, was added to grazoprevir and elbasvir in a triple therapy during the 12-week treatment period after transplantation. All patients achieved a SVR12 and no treatment-related adverse events had occurred [91].

Goldberg et al. [92] evaluated safety and efficacy of kidney transplantation from HCV genotype 1 donors into HCV-negative recipients, followed by elbasvir/grazoprevir treatment in an open-label, single-group, pilot trial. Patients undergoing dialysis who had long awaited kidney transplant have been included in the study and transplanted. Ten patients received HCV-infected kidneys, became HCV RNA positive beginning from the day 3 after transplantation, received a 12-week elbasvir/grazoprevir combination, and achieved SVR12. The authors concluded that the transplantation of HCV genotype 1 infected kidneys into HCV-negative recipients, followed by the administration of elbasvir/grazoprevir, ensures an excellent allograft and the healing of the provoked HCV infection [92].

## 3. Strategies, Future Expectation, and Conclusion on the DAA Treatment of Renal Recipients with HCV Infection

The DAA regimens for HCV-positive renal transplant recipients should be adequate to HCV genotype, the entity of liver disease, the quality of posttransplant renal function, and the immunosuppressive drugs administered. A close monitoring of renal function during the DAA administration, with adjustment of DAA doses to the patients' eGFR, and a careful observation of a possible onset of drug to drag interactions, with adjustment of doses of immunosuppressive drugs, were recommended. For example, sofosbuvir-based DAA regimens may induce renal adverse events in patients with reduced renal function, particularly in those in an end-stage renal disease (ESRD) [93, 94], while the administration of the combination therapy of dasabuvir, ombitasvir, paritaprevir, and ritonavir [95] may lead to drug to drug interactions in patients under a CNI-based immunosuppressive therapy.

Calcineurin inhibitors (tacrolimus and ciclosporin) and the mechanized target of rapamycin inhibitors (everolimus and sirolimus), used as immunosuppressants in patients undergoing renal transplantation, are substrates of cytochrome P450 and therefore the possible interactions between these drugs and DAAs should always be taken into consideration [58, 96–98]. During the DAA treatment of HCV-positive kidney recipients, the decrease in HCV load is frequently followed by a decrease in tacrolimus levels that often requires dose adjustment. This possibility has been well demonstrated by Kamar et al. [66] in renal transplant patients treated with sofosbuvir in various combinations with other antiviral drugs. Although no episodes of rejection or deterioration of the transplanted organ had occurred, the data highlight the need for careful monitoring of immunosuppressive drug levels and dose adjustment [66].

The optimal timing of HCV therapy (pretransplant versus posttransplant) in HCV-positive transplant recipients is topic of debate. Several studies have shown that the wait times for kidney transplantation from HCV-positive donors are significantly shorter than those for kidneys from uninfected donors. These considerations support the use of all kidneys available for transplantation in HCV-positive patients, both HCV-negative and HCV-positive.

The Kidney Disease Improving Global Outcomes guidelines currently recommend treating with DAAs the HCV RNA positive candidates for renal transplant while still in dialysis [41], since once SVR12 has been achieved, a relapse after renal transplant seems unlikely [99]. However, once SVR12 has been achieved with DAA treatment before transplantation, the possibility to receive a renal graft from an HCV-positive donor is lost, together with the benefit of shortening the length of dialysis and of wait time in the kidney transplantation list and with the possibility of reducing comorbidities (i.e., cardiovascular complications) [99, 100]. However, for HCV-positive patients with mild or moderate hepatic fibrosis on a waiting list the high effectiveness of the new DAAs has allowed starting treatment immediately after transplantation [99, 100], a benefit balanced in some cases by the risk of an acute renal injury due to the potential interaction between DAAs and immunosuppressants. In this regard it should also be considered that many of the current DAAs require a creatinine clearance level of 30 ml/min or greater because their main elimination route is renal [58].

Because of the persistent shortage of kidneys that can be transplanted, it has been proposed to transplant HCV-positive kidneys into HCV-negative recipients and to initiate treatment with DAA immediately after transplantation, before the liver disease develops. However, although a few preliminary studies have provided encouraging results, the ethical implications of this procedure should be carefully evaluated, since approximately 5% of patients may not obtain SVR12 with DAA treatment; in some cases the donor HCV genotype may be difficult to treat one (i.e., HCV genotype 3) and in very rare cases fibrosing cholestatic hepatitis may develop after kidney transplantation.

## 4. Final Remarks

A careful assessment of the stage of liver disease should be made including an accurate anamnesis, complete physical examination, serial determinations of serum liver function tests (serum levels of albumin, bilirubin and liver enzymes, prothrombin time, and platelet count), upper abdominal ultrasound examination, and endoscopic examination of the upper intestinal tract in cirrhotic patients. Patients with advanced fibrosis should be regularly revaluated.

The occurrence of reactivation of an indolent (open or occult) Hepatitis B virus (HBV) infection in HBV/HCV kidney‐transplant recipients [77] during or after DAA [76] should be prevented by a nucleoside/nucleotide analogue prophylaxis in HBsAg-positive patients and early identified by an accurate monitoring (ALT serum levels and HBsAg) in HBsAg-negative/anti-HBc-positive patients [74]. In case of HBV reactivation in HBsAg-negative/anti-HBc-positive patients a treatment with nucleoside/nucleotide analogues should be started.

Most DAA therapeutic regimens are practically restricted to HCV genotypes 1 and 4, while HCV genotype 3 is quite frequent and equally dangerous. This need will be overcome by the newer pan-genotypic DAAs regimens (glecaprevir/pibrentasvir).

The optimal regimens to be applied either before or after kidney transplantation may not be proposed yet, because of the expected arrival of other newer DAAs and of results from multicenter randomized controlled trials. Newer DAA regimens requiring a shorter duration of treatment may also accelerate the access to transplantation.

The possibility to transplant HCV-positive kidneys into HCV-negative recipients has been recently analyzed by the American Society of Transplantation Consensus Conference on HCV-positive donors and kidney transplantation. It has been considered that the high level of safety and efficacy of DAAs in eradicating HCV infection provides the opportunity to explore their use in transplanting kidneys from HCV-viremic patients into non-HCV-viremic recipients, a practice that could save the life of numerous CKD patients with organ failure. Although more research is needed before making a final settlement on this topic, the consensus underlines the need to guarantee the access to DAA therapy to the HCV-negative recipients at the time they will receive a kidney from an HCV-positive donor.

It has been also underlined that a living donor who has cleared HCV infection after DAA treatment does not transmit HCV infection to the kidney recipients [101–105].

Treating HCV infection before kidney transplantation could be potentially advantageous, since eradicating HCV would lead to better allograft and patient survival.

Ideally, patients with ESRD and HCV infection should be comanaged by surgeons, nephrologists, and hepatologists before and after transplantation.
