# Erratum to “Eradication of HCV Infection with the Direct-Acting Antiviral Therapy in Renal Allograft Recipients”

**DOI:** 10.1155/2019/8797329

**Published:** 2019-07-15

**Authors:** Armando Calogero, Evangelista Sagnelli, Massimiliano Creta, Silvia Angeletti, Gaia Peluso, Paola Incollingo, Maria Candida, Gianluca Minieri, Nicola Carlomagno, Concetta Anna Dodaro, Massimo Ciccozzi, Caterina Sagnelli

**Affiliations:** ^1^Department of Advanced Biomedical Sciences, University of Naples Federico II, Naples, Italy; ^2^Department of Mental Health and Public Medicine, University of Campania Luigi Vanvitelli, Naples, Italy; ^3^Department of Neurosciences, Human Reproduction and Odontostomatology, University of Naples Federico II, Naples, Italy; ^4^Unit of Clinical Laboratory Science, University Campus Bio-Medico of Rome, Rome, Italy; ^5^Unit of Medical Statistic and Molecular Epidemiology, University Campus Bio-Medico, Rome, Italy

In the article titled “Eradication of HCV Infection with the Direct-Acting Antiviral Therapy in Renal Allograft Recipients” [1], the first and last names of all the authors were reversed. The revised authors' list is shown above and updated in place.

## References

[1] Calogero A., Sagnelli E., Creta M. (2019). Eradication of HCV infection with the direct-acting antiviral therapy in renal allograft recipients. *BioMed Research International*.

